# Evaluation of Hemodynamic Changes in Normovolemic Hypotensive Dogs Treated With Fluids Alone or in Combination With Ephedrine or Dobutamine

**DOI:** 10.1002/vms3.70708

**Published:** 2025-11-27

**Authors:** Pardis Varzandeh, Bahman Mosallanejad, Hadi Imani Rastabi, Mohammad Razi Jalali, Seyed Reza Fatemi Tabatabaei

**Affiliations:** ^1^ Department of Clinical Sciences Faculty of Veterinary Medicine Shahid Chamran University of Ahvaz Ahvaz Iran; ^2^ Department of Basic Sciences Faculty of Veterinary Medicine Shahid Chamran University of Ahvaz Ahvaz Iran

**Keywords:** dobutamine, dog, ephedrine, fluid therapy, hypotension

## Abstract

**Background:**

Normovolemic hypotension occurs due to vasodilation or loss of vascular tone in conditions such as distributive or neurogenic shock. Studies have shown that fluid therapy alone is insufficient in restoring blood pressure in normovolemic hypotension, necessitating the use of complementary drugs.

**Objectives:**

This study aimed to evaluate the effects of fluid therapy with Ringer's solution combined with ephedrine or dobutamine in normovolemic dogs undergoing hypotension induced by isoflurane anaesthesia.

**Methods:**

Anaesthesia in 29 mixed‐breed dogs was induced with propofol (6 mg/kg) titration and maintained with inspired 1.5% isoflurane in 100% oxygen. A total of 15 min after instrumentation (30 min), hypotension (mean arterial blood pressure [MAP] below 60 mmHg) was induced by 3% isoflurane, confirming a direct MAP reading below 60 mmHg for 10 min. Dogs were given one of five treatments of Ringer's solution (1 mL/kg/min, *n* = 5), Ringer's plus ephedrine (0.2 mg/kg, *n* = 6), Ringer's plus dobutamine (5 µg/kg/min, *n* = 6), ephedrine (dosage 0.2 mg/kg, *n* = 6) and dobutamine (5 µg/kg/min, *n* = 6). If direct MAP reached over 60 mmHg and was maintained for 10 min, treatment was discontinued, and the data were recorded. If treatment did not achieve the target of 60 mmHg within 15 min, it was continued for another 15 min. Following the second attempt, drug administration was halted after another 15 min, regardless of the direct MAP level, and the data were recorded. Cardiorespiratory (heart rate, blood pressure, central venous pressure, end tidal CO_2_, SPO_2_ and rectal temperature) and blood gas values were monitored across multiple time points.

**Results:**

Three dogs in the R, two in the D and one in the RE, RD and E treatments needed re‐administration of therapy. Three dogs in R and one in RD and D did not show direct MAP > 60 mmHg after re‐treatment until the isoflurane concentration decreased. After treatment, heart rate was significantly higher in Ringer's plus dobutamine than in dobutamine (*p* = 0.007) and ephedrine (*p* = 0.016). Respiratory and metabolic acidosis were observed in all treatments.

**Conclusions:**

While ephedrine and dobutamine improved hemodynamic variables, crystalloid fluid therapy alone was ineffective in managing normovolemic hypotension in dogs. Future studies with larger samples must assess the combined promising effects of fluid therapy and ephedrine.

## Graphical abstract

Crystalloid fluids alone did not improve normovolemic hypotension in anesthetized dogs. Ephedrine or dobutamine reversed hemodynamic changes, but combining either with fluids offered no added benefit. Notably, dobutamine with fluids may have adverse effects. Larger studies are needed to assess differences between treatments.

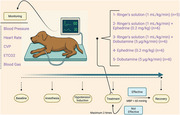

## 1 Introduction

The inadequate delivery of oxygen to meet tissue metabolic demands, termed shock, directly affects blood pressure through cardiac output (CO) and systemic vascular resistance (Herdt et al. 2013). Hypotension, defined as a systolic blood pressure below 80 mmHg and a mean arterial blood pressure (MAP) below 60 mmHg (Muir et al. 2014), poses a critical care challenge, requiring immediate and effective intervention. In veterinary practice, maintaining an MAP of 60 to 70 mmHg is widely acknowledged as essential for ensuring sufficient blood flow to vital organs (Klabunde 2021).

Among the primary causes of hypotension in animals, the loss of body fluids and decreased circulating blood volume (hypovolemia) predominate, although other factors can also contribute to reduced blood pressure (Haskins 2012; Klabunde 2021). Additionally, relative hypovolemia or normovolemic hypotension arising from vasodilation or loss of vascular tone constitutes an often overlooked yet significant cause of hypotension (Summers et al. 2013). The aetiology of normovolemic hypotension due to systemic vasodilation encompasses various conditions such as sepsis, anaphylaxis, autonomic dysfunction, and trauma, all falling under distributive shock (Silverstein and Hopper 2014).

In the clinical management of hypotensive patients, fluid therapy emerges as a cornerstone for improving oxygen supply, restoring blood pressure, and augmenting CO by optimizing preload (Pardo et al. 2024). While fluid administration remains an essential strategy for addressing hypotension resulting from fluid loss, its efficacy in normovolemic animals has spurred debate, accompanied by potential risks, including administering more than the liquid limit (Harold et al. 2013; Valverde et al. 2012).

When fluid resuscitation fails to alleviate hypotension, vasopressor therapy becomes imperative to sustain adequate MAP and tissue perfusion (Armstrong et al. 2017). Ephedrine, a noncatecholamine sympathomimetic with alpha‐ and beta‐adrenergic stimulatory effects, has effectively managed anaesthesia‐induced hypotension in small animals and horses (Chen et al. 2007). Noteworthy benefits of ephedrine therapy include improvements in mean arterial pressure, CO and stroke volume without inducing arrhythmias, thus underscoring its value in addressing hypotensive states (Chen et al. 2007; Trumbull et al. 2021). Similarly, dobutamine, characterized by potent beta‐1 adrenergic agonism and weak alpha‐1 and beta‐2 adrenergic activities, has shown inotropic effects, primarily augmenting stroke volume and CO in normovolemic dogs, with minimal impact on vascular resistance (Dubin et al. 2017; Skelding and Valverde 2020). The dose‐dependent effects of dobutamine on cardiovascular function highlight its potential as a therapeutic option in managing hypotension in veterinary patients (Goya et al. 2018).

No studies have investigated the combined effects of fluid therapy and inotropes or vasopressors in normovolemic dogs experiencing hypotension. Therefore, the objective of the present study was to assess the impact of Ringer's solution administered with or without ephedrine or dobutamine on normovolemic dogs with hypotension induced by isoflurane inhalation anaesthesia. We hypothesized that combining fluid therapy and vasoactive medications would significantly stabilize blood pressure in normovolemic dogs under hypotensive conditions.

## 2 Materials and Methods

*Animals*: Before the commencement of the study, an ethical code (EE/1401.2.24.99424/SCU.ac.ir) was obtained from the Research Ethical Committee of the Shahid Chamran University of Ahvaz. A total of 29 (25 male and four female) 1.5‐ to 2.5‐year‐old male and female dogs of mixed breeds, weighing 20.1 ± 4.3 kg, were transferred to our veterinary hospital and housed in separate cages. They were fed twice daily and had free access to water. The animals' health status was confirmed through a complete clinical examination and a complete blood count test. Their heart sounds and blood pressure were normal prior to the challenge.

*Anaesthesia*: On the examination day, the animals were transferred to the study place and kept for 30 min to acclimate to the environment. Then, the right and left cephalic veins (right for injecting fluids and left for administering drugs) were catheterized with a 20‐gauge intravenous (IV) catheter. The dogs were transferred to a surgery table and anaesthetized with titration of propofol (6 mg/kg; 1%, Braun, Melsungen, Germany). After intubation with an appropriate cuffed endotracheal tube (8–8.5 mm internal diameter), the dogs were placed in the right lateral recumbency and connected to a circle rebreathing anaesthetic circuit. The dogs were then maintained under anaesthesia by isoflurane (Forane, Abbott, UK) in 100% oxygen, with an oxygen flow rate of 2 L/min. Volume‐controlled mechanical ventilation was performed with a breathing rate of 8–10/min and a tidal volume of 10–15 mL/kg to maintain end‐tidal CO_2_ (ETCO_2_) at 35–45 mmHg. A heating pad was used to keep the central body temperature at 37°C–38°C. Ringer's solution (Iranian Parenteral and Pharmaceutical Co., Tehran, Iran) was infused with a 1 mL/kg/h maintenance dose rate.

*Instrumentation*: The dorsal pedal artery was surgically exposed, catheterized using a 22‐gauge angiocat polyflon catheter (Polyflon, Afra Teb, Iran), and connected to an aneroid manometer (ALPK2, Japan) for continuous direct MAP monitoring and anaerobic collection of arterial blood samples (1 mL per time). To ensure accurate blood pressure measurements, the transducer was carefully positioned at the heart level, aligned with the external sternal notch. Regular checks for resonance and stability were conducted during the experiment to prevent any potential inaccuracies in measurements. Additionally, the transducer was maintained in a secure position throughout the procedure to minimize movement‐related errors. The jugular vein was also catheterized with a central venous pressure (CVP) measurement apparatus (Arrow International, PA, USA) using the Selndiger technique. The position of the jugular catheter tip was confirmed by radiography and aspirating blood to verify correct placement in the right atrium. The electrodes of the electrocardiograph (Guangdong Biolight Meditech Co, BLT‐1203B, China) were connected to the skin of the elbow and knee areas of the animal, and the junction was moistened with alcohol. An electrocardiogram (ECG) was taken continuously at a speed of 50 mm/s and a voltage of 10 mV. The dogs were also connected to a multiparameter monitoring system (Burtons, PM‐9000Vet, UK) to measure oxygen saturation of haemoglobin (SPO_2_), heart rate (HR), non‐invasive blood pressure (NIBP, using a cuff with at least 40% circumference of the metatarsal area aligned to the heart level indicated by the external sternal notch), respiratory rate (*f*_R_), rectal temperature (RT), and ETCO_2_. All the necessary instruments were utilized to perform the procedures within 30 min of administering anaesthesia.

*Experiment*: After instrumentation, the dogs were maintained under general anaesthesia with isoflurane 1.5% for an additional 15 min. Data were recorded as the baseline of the study (Time 0). Then, the depth of anaesthesia using 3% isoflurane was increased until the mean direct blood pressure reached below 60 mmHg (hypotension) and remained in this state for 10 min. At this point, the data were measured and recorded (T1). Then, dogs were randomly divided into five groups (https://www.randomizer.org/). The first group received the Ringer's solution (R, *n* = 5) at a 1 mL/kg/min rate for a maximum of 15 min. In the second group, the Ringer's solution (1 mL/kg/min) was administered in conjunction with intravenous (IV) ephedrine (HBM Pharma s.r.o., Sklabinska Martin, Slovak Republic) at 0.2 mg/kg (RE, *n* = 6). In the third group, Ringer's solution (1 mL/kg/min) with constant rate infusion (CRI) of dobutamine (Hameln Pharma GmbH Inselstrabe, Hameln, Germany) at a dose of 5 µg/kg/min was administered (RD, *n* = 6). In the fourth and fifth groups, IV administration of ephedrine (E, *n* = 6) and CRI of dobutamine (D, *n* = 6) with the same dose as before were performed, respectively. If, after treatment, the direct MAP reached over 60 mmHg and was maintained for 10 min, the administration of drugs was discontinued, and the data were recorded (T2). Otherwise, the treatment would be repeated, and regardless of the direct MAP level, the data would be recorded 15 min later (T2). After that, the administration of drugs was discontinued. After discontinuing the treatment administration, the isoflurane concentration was returned to 1.5% isoflurane within 15 min, and the data were recorded (T3). Afterward, the isoflurane concentration was reduced gradually to zero within 15 min, and the dogs were allowed to recover. The catheters were removed after the final data collection time point (T3). Post‐anaesthetic pain in dogs was clinically assessed daily for 1 week using indicators such as heart rate, respiratory rate, vocalization, and behaviour. Dogs showing signs of pain were provided treatment with IV administration of ketoprofen (1.1 mg/kg).

*Assessments*: At each time point, HR, direct MAP, NIBP (indirect systolic, diastolic, and MAP; SBP, DAP and MAP, respectively), CVP (to surrogate for preload/venous return and right‐sided filling pressures), ETCO_2_, SPO_2_ and RT were recorded. Using a blood gas analyser (EDAN i15), pH, PCO_2_ (mmHg), PO_2_ (mmHg), bicarbonate (${\mathrm{HCO}}_{3}^{-}$, mmol/L) concentration and base excess (BE, mmol/L) were determined as tissue perfusion adequacy and metabolic status indicators. ECG was used to monitor any potential dysrhythmias.

### 2.1 Statistical Analysis

Data were analysed using GraphPad Prism 9.0.0 and Excel 2016. The normal distribution of the data was confirmed using the Shapiro–Wilk test. A one‐way ANOVA with Bonferroni post hoc test was used to compare the data between treatments. A mixed model and Bonferroni post hoc test were also used to compare the data over time. Data are shown as mean ± standard deviation (SD), and *p* < 0.05 was considered significant.

## 3 Results

All dogs tolerated the anaesthesia and hypotension processes well and successfully recovered. No death or complications were seen related to the study's procedures and a 2‐week follow‐up. There was no significant difference in the weight of the studied dogs and the amount of propofol consumed at induction between the animals of the five studied groups (*p* > 0.05, Table 1).

**TABLE 1:** Mean ± standard deviation of weight and propofol consumption in isoflurane‐induced hypotensive dogs treated with either (1) Ringer's solution (R, *n* = 5), (2) Ringer's solution with intravenous (IV) administration of ephedrine (RE, *n* = 6), (3) Ringer's solution with IV administration of dobutamine (RD, *n* = 6), (4) IV administration of ephedrine (E, *n* = 6) or (5) constant rate infusion of dobutamine (D, *n* = 6).

Parameters	R	RE	RD	E	D
Weight (kg)	22.1 ± 4.8	19.9 ± 6.0	21.0 ± 3.7	17.3 ± 2.2	20.7 ± 4.3
Propofol consumption (mL/kg)	11.8 ± 3.2	9.0 ± 2.7	11.8 ± 3.3	11.0 ± 2.0	12.2 ± 3.1

Three dogs out of five in the R treatment, two out of six in the D treatment, and one in the RE, RD and E treatments needed re‐administration of therapy. Three dogs in the R and one dog in the RD and D treatments did not reach the target of direct blood pressure above 60 mmHg after re‐treatment until the isoflurane concentration decreased. Once the direct blood pressure was increased above 60 mmHg and maintained for 15 min, a subsequent drop below 60 mmHg was not observed in any of the treated animals.

Heart rate was significantly higher in RD than in D (*p* = 0.007) and E (*p* = 0.016) at T2. The within‐group comparison showed that the HR in the RD was significantly higher than the baseline at T2 (*p* = 0.028). For NIBP, MAP in all treatments, except for R, was significantly lower at T2 than the baseline (*p* ≤ 0.020). It was also significantly lower in E at T2 (*p* = 0.020) and D at T3 (*p* = 0.010) compared to the baseline. Systolic and diastolic blood pressure in the D (*p* = 0.002) and RD (*p* = 0.001) at T1 were significantly lower than the baseline values, respectively. Direct MAP was significantly lower than the baseline at T1 in RE and D treatments (*p* = 0.046, *p* = 0.037, respectively). The CVP was significantly lower at baseline than T2 in R (*p* = 0.040) and T1 and 2 in RD (*p* = 0.009, *p* = 0.020, respectively) (Table 2).

**TABLE 2:** Mean ± standard deviation of hemodynamic parameters in isoflurane‐induced hypotensive dogs treated with either (1) Ringer's solution (R, *n* = 5), (2) Ringer's solution with intravenous (IV) administration of ephedrine (RE, *n* = 6), (3) Ringer's solution with IV administration of dobutamine (RD, *n* = 6), (4) IV administration of ephedrine (E, *n* = 6) or (5) constant rate infusion of dobutamine (D, *n* = 6); T1: 10 min of being in hypotensive state; T2: after treatment; T3: recovery.

Parameter	Group	Baseline	T1	T2	T3
Heart rate (beats/min)	R	117 ± 23	100 ± 20	103 ± 20	105 ± 16
	RE	110 ± 13	88 ± 41	110 ± 26	125 ± 44
	RD	108 ± 28	92 ± 12	184 ± 15 ^bA^	119 ± 19
	E	113 ± 13	95 ± 18	99 ± 24 ^c^	110 ± 38
	D	112 ± 31	107 ± 12	108 ± 20 ^c^	114 ± 18
Direct arterial blood pressure (mmHg)	R	83 ± 24	44 ± 5	46 ± 12	60 ± 17
	RE	87 ± 25	41 ± 6 ^A^	87 ± 30	72 ± 23
	RD	73 ± 10	38 ± 15	71 ± 14	56 ± 5
	E	66 ± 16	40 ± 6	67 ± 11	66 ± 19
	D	84 ± 25	37 ± 8 ^A^	61 ± 13	61 ± 12
Indirect mean arterial blood pressure (mmHg)	R	77 ± 22	45 ± 11	52 ± 12	60 ± 10
	RE	77 ± 21	42 ± 7 ^A^	75 ± 21	76 ± 12
	RD	75 ± 13	44 ± 10 ^A^	69 ± 22	66 ± 11
	E	73 ± 10	43 ± 6 ^A^	62 ± 6 ^A^	63 ± 4
	D	87 ± 17	43 ± 6 ^A^	62 ± 16	70 ± 7 ^A^
Indirect systolic blood pressure (mmHg)	R	107 ± 31	70 ± 12	76 ± 17	87 ± 20
	RE	119 ± 26	68 ± 8	132 ± 40	110 ± 17
	RD	101 ± 12	65 ± 20	99 ± 22	85 ± 10
	E	98 ± 24	67 ± 8	99 ± 13	92 ± 14
	D	115 ± 25	62 ± 10 ^A^	100 ± 14	92 ± 10
Indirect diastolic blood pressure (mmHg)	R	60 ± 25	34 ± 10	31 ± 12	42 ± 13
	RE	62 ± 25	24 ± 4	62 ± 34	53 ± 24
	RD	60 ± 10	32 ± 10 ^A^	50 ± 13	41 ± 6
	E	49 ± 18	26 ± 8	47 ± 7	44 ± 4
	D	62 ± 24	24 ± 5	47 ± 18	46 ± 20
Central venous pressure (cmH2o)	R	11 ± 1	12 ± 2	15 ± 3 ^A^	13 ± 3
	RE	11 ± 3	12 ± 3	13 ± 3	10 ± 2
	RD	9 ± 2	14 ± 2 ^A^	16 ± 3 ^A^	12 ± 1
	E	11 ± 5	14 ± 5	15 ± 6	12 ± 5
	D	9 ± 2	12 ± 5	12 ± 5	10 ± 3

*Note*: Superscript capital letters show significant differences compared to the baseline (*p* < 0.05). Superscript lowercase letters show significant differences among treatments (*p* < 0.05).

Blood gas analysis revealed that pH was significantly lower at T2 and T3 compared to the baseline in the RE (*p* = 0.037, *p* = 0.023, respectively) and E (*p* = 0.020, *p* = 0.033, respectively). PCO_2_ significantly differed at T2 compared to the baseline in the D treatment (*p* = 0.002) (Table 3).

**TABLE 3:** Mean ± standard deviation of blood gas parameters in isoflurane‐induced hypotensive dogs treated with either (1) Ringer's solution (R, *n* = 5), (2) Ringer's solution with intravenous (IV) administration of ephedrine (RE, *n* = 6), (3) Ringer's solution with IV administration of dobutamine (RD, *n* = 6), (4) IV administration of ephedrine (E, *n* = 6) or (5) constant rate infusion of dobutamine (D, *n* = 6); T1: 10 min of being in hypotensive state; T2: after treatment; T3: recovery.

Parameter	Group	Baseline	T1	T2	T3
Arterial blood pH	R	7.30 ± 0.10	7.25 ± 0.07	7.21 ± 0.07	7.26 ± 0.04
	RE	7.32 ± 0.06	7.21 ± 0.11	7.18 ± 0.12 ^A^	7.24 ± 0.08 ^A^
	RD	7.30 ± 0.07	7.26 ± 0.11	7.17 ± 0.11	7.23 ± 0.09
	E	7.36 ± 0.10	7.31 ± 0.14	7.28 ± 0.10 ^A^	7.30 ± 0.09 ^A^
	D	7.32 ± 0.03	7.27 ± 0.03	7.18 ± 0.04	7.25 ± 0.02
PO_2_ (mmHg)	R	490 ± 136	459 ± 192	468 ± 224	480 ± 136
	RE	410 ± 153	318 ± 258	329 ± 222	378 ± 153
	RD	549 ± 134	510 ± 161	537 ± 230	531 ± 116
	E	613 ± 70	578 ± 224	605 ± 55	588 ± 136
	D	592 ± 122	529 ± 192	547 ± 210	530 ± 125
PCO_2_ (mmHg)	R	41 ± 2	51 ± 113	52 ± 14	55 ± 3
	RE	46 ± 6	52 ± 4	52 ± 13	63 ± 7
	RD	46 ± 3	48 ± 8	47 ± 6	46 ± 3
	E	48 ± 12	51 ± 14	49 ± 15	48 ± 9
	D	37 ± 7	50 ± 11	58 ± 3 ^A^	50 ± 6
Bicarbonate (mmol/L)	R	24 ± 2	22 ± 2	20 ± 2	25 ± 4
	RE	27 ± 3	25 ± 4	23 ± 4	24 ± 5
	RD	26 ± 4	25 ± 3	24 ± 4	25 ± 3
	E	26 ± 2	25 ± 3	26 ± 4	26 ± 2
	D	25 ± 4	24 ± 3	22 ± 3	23 ± 5
Base excess (mmol/L)	R	0 ± 1	−3 ± 2	−5 ± 2	−1 ± 4
	RE	0 ± 5	−1 ± 3	−3 ± 2	−2 ± 5
	RD	0 ± 4	−2 ± 4	−5 ± 3	−4 ± 3
	E	0 ± 2	−2 ± 2	−1 ± 3	0 ± 1
	D	−2 ± 3	−3 ± 2	−4 ± 2	−3 ± 4

*Note*: Superscript capital letters show significant differences compared to the baseline (*p* < 0.05).

ECG assessments revealed that sinus tachycardia arrhythmias in RD and RE, and sinus bradycardia in E, R and RE treatments were observed; sinus arrest and wandering pacemaker in R and E treatments were observed, respectively; premature ventricular contraction in RD treatment and ST‐segment elevation in R, RE and E treatments were observed. The heart rhythm in the rest of the ECG recording cases was sinus rhythm or sinus arrhythmia.

## 4 Discussion

In this study, we investigated the physiological responses following the induction of normovolemic hypotension by reducing blood pressure to below 60 mmHg and maintaining this hypotensive state for 10 min by administering a high concentration of isoflurane in canines. According to Chen et al. (2007), normovolemic hypotension induced by isoflurane anaesthesia is characterized by a reduction in blood pressure attributed to vasodilation, decreased myocardial contractility and alterations in HR. Supporting this, Yang et al. (2014) reported that increased concentrations of isoflurane lead to significant reductions in HR, myocardial contractility, MAP and systolic and diastolic function of the left ventricle. In our study, we observed a downward trend in HR, direct and indirect MAP 10 min after increasing the isoflurane concentration from 1.5% to 3%. As our objective was to maintain direct MAP below 60 mmHg for a minimum of 10 min, and all experimental subjects successfully achieved this target, the model utilized proved effective for inducing normovolemic hypotension in dogs.

The data obtained in the present study demonstrated that fluid therapy alone using the Ringer's solution did not improve HR or blood pressure in normovolemic hypotensive dogs induced by high dose of isoflurane. Specifically, direct blood pressure remained below 60 mmHg in 3 out of 5 dogs (i.e., 60%) following the initial administration of the Ringer's solution and did not increase to 60 mmHg after a second administration, indicating the ineffectiveness of fluid therapy alone in enhancing blood pressure. Notably, CVP increased significantly compared to baseline in this group, showing that this increase does not necessarily correlate with an improvement in blood pressure. Indeed, CVP reflects preload, but it can also be influenced by factors like afterload, HR and myocardial contractility. In the current study, increased CVP despite no improvement in BP can be attributed to the inability of the cardiovascular system to effectively convert this volume into arterial blood pressure due to vasodilation, resulting in blood pressure not rising accordingly. This could also reflect decreased myocardial contractility and CO that occurs with high doses of isoflurane, leading to ineffective pumping of the administered fluid into the systemic circulation.

The cardiovascular effects of ephedrine (0.1 mg/kg) are reported to share characteristics with those of norepinephrine and epinephrine, including an increase in blood pressure, enhanced myocardial contractility, a rapid but modest rise in HR and peripheral vasoconstriction (Chen et al. 2007). Alsufyani and Docherty (2018) observed that ephedrine induces a dose‐dependent increase in heart rate and blood pressure. Trumbull et al. (2021) also found that ephedrine (0.05–0.1 mg/kg) effectively raises blood pressure in anaesthesia‐associated hypotension in pinnipeds. In the present study, bolus administration of ephedrine (0.1 mg/kg) resulted in significant increases in direct and indirect MAP without substantial changes in HR. It has been stated that since the indirect effect of ephedrine (production of norepinephrine) on arterial blood pressure and the action of vasoconstriction is done chiefly on the venous system, ephedrine is effective in raising the CVP when the patient faces a fluid volume disorder (Skelding and Valverde 2020). Our results show that although CVP in the E and RE groups increased slightly after therapy, the changes were insignificant. Aarnes et al. (2009) reported that the administration of Ringer's lactate solution did not significantly affect HR or arterial blood pressure, leading to the conclusion that it is not recommended for treating hypotension induced by isoflurane in dogs.

In the current study, dobutamine was administered at a dose of 5 µg/kg/min, which is consistent with the dosage used by Dyson and Sinclair (2006) (i.e., 3–6 µg/kg/min) in their study of hypovolemic dogs. Administration of dobutamine with the mentioned dose rate, in this study, did not yield significant changes in HR compared to baseline levels in hypovolemic dogs. However, the addition of concurrent fluid therapy to dobutamine infusion resulted in a tremendous increase in HR in the fluid therapy group (RD) compared to the dobutamine (D) and ephedrine (E), which may negatively result in a greater oxygen demand for the heart muscle. The HR increase in the RD group may reflect preload augmentation from fluid therapy, triggering reflex sympathetic activation or reduced vagal tone. In addition, dobutamine's β1‐adrenergic action, combined with increased preload, can shift the haemodynamic balance toward a higher HR to maintain CO, and altered pharmacokinetics/distribution with fluids might have amplified this effect. Regarding blood pressure, dobutamine with and without fluid therapy showed no significant changes compared to baseline and other treatment modalities. Nonetheless, direct and indirect MAP values were slightly elevated in the RD. Several studies have reported that the administration of dobutamine in healthy dogs anaesthetized with isoflurane increases CO without a corresponding increase in arterial pressure. For instance, Dyson and Sinclair (2006) showed that the administration of dobutamine (at a dose of 3–6 µg/kg/min) in hypovolemic dogs under isoflurane anaesthesia did not affect blood pressure but increased HR, cardiac index and stroke volume. These effects are attributed to dobutamine's inotropic and vasodilatory properties (Rosati et al. 2007). Sousa et al. (2005) investigated the dobutamine stress test in five healthy, conscious dogs and found that HR increased in a dose‐dependent manner. Dubin et al. (2017) also noted that dobutamine primarily enhances stroke volume and CO through its inotropic effects while causing only a modest increase in HR.

Blood acidity increased in all groups following the induction of hypotension. Despite treatment, this downward trend in blood pH persisted. Additionally, the partial pressure of carbon dioxide (P_a_CO_2_) rose after hypotension was induced, suggesting the presence of respiratory acidosis. This respiratory acidosis continued into the recovery period, indicating that lung and tissue perfusion were still insufficient due to hypovolemia and hypotension during anaesthesia. Interestingly, BE also exhibited a decreasing trend throughout the experiment, suggesting a concurrent occurrence of respiratory and metabolic acidosis in normovolemic hypotensive dogs, potentially resulting from diminished tissue oxygenation (Bach 2017).

This study has several limitations. First, one experimental group (Ringer's group, *n* = 5) contained one fewer subject than the others (*n* = 6) due to the exclusion of a pregnant dog and practical constraints that prevented replacement. While this minor imbalance in group sizes could introduce marginal variability, its impact on the overall results is likely negligible given the consistency of our experimental protocols and the robustness of the observed effects. Second, using vaporizer settings (1.5% isoflurane dial concentration) instead of direct measurement of end‐tidal isoflurane (ETIso) represents another limitation. Although vaporizer settings offer a standardized and clinically practical method for anaesthetic delivery, they do not account for individual variability in drug uptake, metabolism or potential equipment performance discrepancies. This limitation is amplified when considering higher anaesthetic depths (e.g., 3% isoflurane). In our study, haemodynamic stability and adequate surgical anaesthesia were reliably achieved at 1.5% isoflurane, but it would be inappropriate to assume identical stability or effects at 3% or other depths. Consequently, concentration‐dependent effects could act as a confounder and may limit the generalizability of our findings across anaesthetic depths. However, we implemented rigorous clinical monitoring of anaesthetic depth (e.g., assessment of reflexes, haemodynamic stability) and ensured all vaporizers were properly calibrated. Third, the relatively small sample size (*n* = 5–6 per group), primarily constrained by financial and logistical considerations, limits the statistical power to detect subtle effects. However, this study was designed as an investigation to identify clinically meaningful trends and generate hypotheses for future research.

## 5 Conclusion

The findings indicate that fluid therapy utilizing crystalloid solutions alone did not enhance cardiovascular function in dogs with normovolemic hypotension induced by high doses of isoflurane. In contrast, administering ephedrine or dobutamine effectively reversed adverse alterations in haemodynamic variables. While the combination of fluid therapy and ephedrine produced comparable outcomes to the administration of ephedrine or dobutamine alone, further studies with a larger sample size are necessary to identify potential significant differences. Notably, the addition of dobutamine to fluid therapy appeared not to exert a beneficial effect, and it might be associated with adverse outcomes.

## Author Contributions

**Pardis Varzandeh**: conceptualization, data curation, writing – original draft. **Bahman Mosallanejad**: conceptualization, investigation, supervision, funding acquisition. **Hadi Imani Rastabi**: conceptualization, data curation, validation, formal analysis, supervision, funding acquisition, project administration, writing – review and editing. **Mohammad Razi Jalali**: methodology, supervision. **Seyed Reza Fatemi Tabatabaei**: conceptualization, methodology, supervision.

## Funding

This study was funded by the Research Council of Shahid Chamran University of Ahvaz (grant number 96791216).

## Conflicts of Interest

The authors declare no conflicts of interest.

## Data Availability

Data are available upon request from the authors.

## References

[vms370708-bib-0001] Aarnes, T. K. , R. M. Bednarski , P. Lerche , J. A. Hubbell , and W. W. Muir . 2009. “Effect of Intravenous Administration of Lactated Ringer's Solution or Hetastarch for the Treatment of Isoflurane‐Induced Hypotension in Dogs.” American Journal of Veterinary Research 70, no. 11: 1345–1353. 10.2460/ajvr.70.11.1345. 19878017

[vms370708-bib-0002] Alsufyani, H. A. , and J. R. Docherty . 2018. “Direct and Indirect Effects of Ephedrine on Heart Rate and Blood Pressure in Vehicle‐Treated and Sympathectomised Male Rats.” European Journal of Pharmacology 825: 34–38. 10.1016/j.ejphar.2018.02.021. 29454610

[vms370708-bib-0003] Armstrong, B. A. , R. D. Betzold , and A. K. May . 2017. “Sepsis and Septic Shock Strategies.” Surgical Clinics 97, no. 6: 1339–1379. 10.1016/j.suc.2017.07.003. 29132513

[vms370708-bib-0004] Bach, J. 2017. “A Quick Reference on Hypoxemia.” In Advances in Fluid, Electrolyte, and Acid‐Base Disorders, an Issue of Veterinary Clinics of North America: Small Animal Practice, edited by H. A . de Morais and S. P . DiBartola , 175–179. Elsevier Health Sciences.

[vms370708-bib-0006] Chen, H. C. , M. D. Sinclair , and D. H. Dyson . 2007. “Use of Ephedrine and Dopamine in Dogs for the Management of Hypotension in Routine Clinical Cases Under Isoflurane Anesthesia.” Veterinary Anaesthesia and Analgesia 34, no. 5: 301–311. 10.1111/j.1467-2995.2006.00327.x. 17532806

[vms370708-bib-0008] Dubin, A. , B. Lattanzio , and L. Gatti . 2017. “The Spectrum of Cardiovascular Effects of Dobutamine‐From Healthy Subjects to Septic Shock Patients.” Revista Brasileira De Terapia Intensiva 29: 490–498. 10.5935/0103-507X.20170068. 29340539 PMC5764562

[vms370708-bib-0009] Dyson, D. , and M. Sinclair . 2006. “Impact of Dopamine or Dobutamine Infusions on Cardiovascular Variables After Rapid Blood Loss and Volume Replacement During Isoflurane Induced Anesthesia in Dogs.” American Journal of Veterinary Research 67, no. 7: 1121–1130. 10.2460/ajvr.67.7.1121. PMID:16817731. 16817731

[vms370708-bib-0010] Goya, S. , T. Wada , K. Shimada , D. Hirao , and R. Tanaka . 2018. “Dose‐Dependent Effects of Isoflurane and Dobutamine on Cardiovascular Function in Dogs With Experimental Mitral Regurgitation.” Veterinary Anaesthesia and Analgesia 45, no. 4: 432–442. 10.1016/j.vaa.2018.03.010. PMID: 29887228. 29887228

[vms370708-bib-0011] Harold, D. , T. Jensen , A. Johnson , P. Knowles , R. Meyer , and R. Rucinsky . 2013. “AAHA/AAFP Fluid Therapy Guidelines for Dogs and Cats.” Journal of American Animal Hospital Association 49, no. 3: 149–159. 10.5326/jaaha-ms-5868. 23645543

[vms370708-bib-0012] Haskins, S. C. 2012. “ *Shock* .” In Manual of Small Animal Emergency and Critical Care Medicine. 2nd ed., edited by D. K. Macintire , K. J. Drobatz , S. C. Haskins , and W. D. Saxon , 30–40. John Wiley & Sons.

[vms370708-bib-0013] Herdt, T. H. , A. I. Sayegh , and B. G. Klein . 2013. Cunningham's Textbook of Veterinary Physiology. Elsevier Health Sciences.

[vms370708-bib-0015] Klabunde, R. 2021. Cardiovascular Physiology Concepts. Wolters Kluwer Publication.

[vms370708-bib-0016] Muir, W. W. , Y. Ueyama , A. Pedraza‐Toscano , et al. 2014. “Arterial Blood Pressure as a Predictor of the Response to Fluid Administration in Euvolemic Nonhypotensive or Hypotensive Isoflurane‐Anesthetized Dogs.” Journal of the American Veterinary Medical Association 245, no. 9: 1021–1027. 10.2460/javma.245.9.1021. 25313813

[vms370708-bib-0017] Nascimento, P., Jr , O. de Paiva Filho , L. R. de Carvalho , and J. R. Braz . 2006. “Early Hemodynamic and Renal Effects of Hemorrhagic Shock Resuscitation With Lactated Ringer's Solution, Hydroxyethyl Starch, and Hypertonic Saline With or Without 6% Dextran‐70.” Journal of Surgical Research 136, no. 1: 98–105. 10.1016/j.jss.2006.04.021. PMID: 16815449. 16815449

[vms370708-bib-0019] Pardo, M. , E. Spencer , A. Odunayo , et al. 2024. “2024 AAHA Fluid Therapy Guidelines for Dogs and Cats.” Journal of the American Animal Hospital Association 60, no. 4: 131–163. 10.5326/JAAHA-MS-7444. PMID: 38885492. 38885492

[vms370708-bib-0021] Rosati, M. , D. H. Dyson , M. D. Sinclair , and W. C. Sears . 2007. “Response of Hypotensive Dogs to Dopamine Hydrochloride and Dobutamine Hydrochloride During Deep Isoflurane Anesthesia.” American Journal of Veterinary Research 68, no. 5: 483–494. 10.2460/ajvr.68.5.483. 17472447

[vms370708-bib-0022] Silverstein, D. , and K. Hopper . 2014. Small Animal Critical Care Medicine. Elsevier Publication.

[vms370708-bib-0024] Skelding, A. M. , and A. Valverde . 2020. “Sympathomimetics in Veterinary Species Under Anesthesia.” Veterinary Journal 258: 105455. 10.1016/j.tvjl.2020.105455. 32564865

[vms370708-bib-0025] Sousa, M. G. , G. B. Pereira‐Neto , R. Carareto , D. G. Gerardi , and A. A. Camacho . 2005. “Assessment of Electrocardiographic Parameters in Healthy Dogs Undergoing Dobutamine Stress Testing: Veränderungen Elektrokardiographischer Parameter bei Gesunden Hunden Während Dobutamin Stressuntersuchungen.” Schweizer Archiv Für Tierheilkunde 147, no. 12: 541–545. 10.1024/0036-7281.147.12.541. 16398192

[vms370708-bib-0026] Summers, R. L. , S. D. Baker , S. A. Sterling , J. M. Porter , and A. E. Jones . 2013. “Characterization of the Spectrum of Hemodynamic Profiles in Trauma Patients With Acute Neurogenic Shock.” Journal of Critical Care 28, no. 4: 531. 10.1016/j.jcrc.2013.02.002. PMC4043212 23566731

[vms370708-bib-0027] Trumbull, E. J. , F. Okonski , C. L. Field , et al. 2021. “The Use of Ephedrine to Treat Anesthesia‐Associated Hypotension in Pinnipeds.” Journal of Zoo and Wildlife Medicine 52, no. 3: 1054–1060. 10.1638/2020-0219. 34687524

[vms370708-bib-0028] Valverde, A. , G. Gianotti , E. Rioja‐Garcia , and A. Hathway . 2012. “Effects of High‐Volume, Rapid‐Fluid Therapy on Cardiovascular Function and Hematological Values During Isoflurane‐Induced Hypotension in Healthy Dogs.” Canadian Journal of Veterinary Research 76, no. 2: 99–108. 23024452 PMC3314442

[vms370708-bib-0029] Yang, C. F. , M. Y. C. Chen , T. I. Chen , and C. F. Cheng . 2014. “Dose‐Dependent Effects of Isoflurane on Cardiovascular Function in Rats.” Tzu Chi Medical Journal 26, no. 3: 119–122. 10.1016/j.tcmj.2014.07.005.

